# Diagnostic and Therapeutic Approaches for a Diabetic Patient Presenting with Secondary Syphilis and Severe Odynophagia

**DOI:** 10.3390/medicina60020298

**Published:** 2024-02-09

**Authors:** Bramantono Bramantono, Henry Sutanto, Hermawan Susanto, Muhammad Vitanata Arfijanto, Usman Hadi

**Affiliations:** 1 Department of Internal Medicine, Faculty of Medicine, Universitas Airlangga, Surabaya 60132, Indonesia; ibambramantono@gmail.com (B.B.); hermawan-s@fk.unair.ac.id (H.S.); muhammad-v-a@fk.unair.ac.id (M.V.A.); usman.hadi@fk.unair.ac.id (U.H.); 2 Division of Tropical and Infectious Diseases, Department of Internal Medicine, Dr. Soetomo General Academic Hospital, Surabaya 60286, Indonesia; 3 Division of Endocrinology, Metabolic Diseases and Diabetes, Department of Internal Medicine, Dr. Soetomo General Academic Hospital, Surabaya 60286, Indonesia

**Keywords:** *Treponema pallidum*, syphilis, odynophagia, infectious disease, diabetes mellitus, sexually transmitted infection

## Abstract

Syphilis, an infectious disease caused by the spirochete *Treponema pallidum*, represents a pervasive global epidemic. Secondary syphilis is typically marked by the emergence of highly contagious mucocutaneous manifestations, including non-pruritic rashes on the palms and soles of the feet, alopecia, mucous patches, and condyloma lata. Here, we report a rare case of a 30-year-old male with newly discovered type 2 diabetes mellitus who presented with severe odynophagia due to secondary syphilis, confirmed by both nontreponemal VDRL/RPR and treponemal TPHA tests. Following the administration of a single-dose intramuscular injection of benzathine penicillin G 2.4 million units, the symptoms gradually decreased, allowing the patient to regain his health.

## 1. Introduction

Syphilis, an infectious disease caused by the spiral-shaped bacterium (spirochete) *Treponema pallidum*, represents a pervasive global epidemic, particularly affecting high-risk populations, such as men who have sex with men. According to the 2020 report from the World Health Organization (WHO), 7.1 million individuals between the ages of 15 and 49 contracted this sexually transmitted disease worldwide. Notably, the majority of these infections are asymptomatic [1]. Syphilis is frequently transmitted from one individual to another through direct skin-to-skin contact during sexual activities with a person exhibiting active primary or secondary lesions [2]. Additionally, alternative routes of syphilis transmission have been documented, including transmission through oral sex, transplacental (congenital) transmission, and blood transfusion [2].

Syphilis infection progresses through distinct stages: primary, secondary, latent, and tertiary syphilis. Primary syphilis typically manifests 10 to 90 days after bacterial exposure. It is characterized by the development of a painless, indurated genital or anal chancre, which usually heals unnoticed within 3–10 days but can persist for 3–6 weeks [1,3]. Without adequate treatment during this stage, the infection can progress to the secondary stage. Secondary syphilis arises 2–8 weeks after the resolution of the initial ulcer (*ulcus durum*) and is marked by the emergence of highly contagious, self-limiting mucocutaneous manifestations. These include non-pruritic rashes on the palms and soles of the feet, alopecia, and mucous patches. Additionally, condyloma lata may develop, characterized by white and grey, flat-topped, well-defined hypertrophic papules or plaques occurring in warm and moist intertriginous areas, such as the labia, genitalia, and anus [1,3,4]. On a rare occasion, secondary syphilis can exhibit renal manifestations, such as glomerulopathies, tubular pathology, and vasculitis in the kidney [5]. Failure to treat secondary syphilis adequately can lead to further disease progression. Latent syphilis is typically asymptomatic and can only be identified through positive serological testing. If left untreated, latent syphilis can advance to tertiary syphilis, the final stage of the disease. Tertiary syphilis often presents as a systemic condition with multiorgan complications, including cardiovascular and neurological complications (i.e., neurosyphilis) [1,3]. 

Diabetes mellitus represents a global health burden, affecting 462 million people, accounting for 6.28% of the world’s population in 2017 [6]. This condition is characterized by a chronic elevation of blood glucose levels, resulting from the failure of pancreatic beta cells to produce an adequate amount of insulin or the ineffective utilization of insulin by cells, a phenomenon referred to as insulin resistance [7]. In addition to its well-known neurological, microvascular, and macrovascular complications, diabetes can also compromise immunity through various mechanisms. These include impaired cytokine production, inhibition of leukocyte recruitment, failure in pathogen recognition, dysfunction of innate immune cells (such as neutrophils, macrophages, and natural killer cells), as well as the suppression of cellular responses (such as reduced T cell activity) and humoral immunity involving antibodies and complements [7,8]. Such impairment of both adaptive and humoral immunity may render individuals more susceptible to infections and potentially exacerbate the prognosis of existing infections [9]. 

In this manuscript, we sought to present a clinical case involving a patient newly diagnosed with type 2 diabetes mellitus (T2DM) who experienced severe odynophagia resulting from extensive oropharyngeal ulcers due to secondary syphilis. Subsequently, we will delve into recommendations for the diagnosis and management of this complex condition.

## 2. Case Illustration

**Figure 1:**
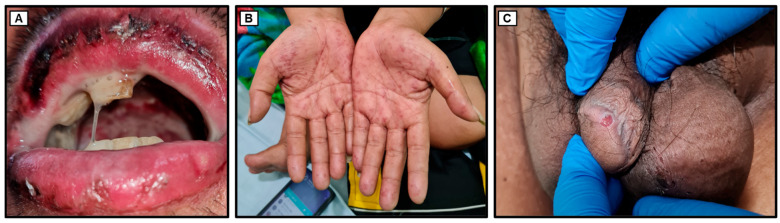
Initial clinical presentation at the Emergency Unit of Dr. Soetomo General Academic Hospital. (**A**) Swollen lips with crusts, accompanied by hypersalivation. Thick white plaques were observed throughout the oral cavity, notably on the hard and soft palate. (**B**) Multiple non-pruritic rashes are present on both hands. (**C**) A painless genital ulcer is located adjacent to the penile glans.

A 30-year-old male presented to the Emergency Unit of Dr. Soetomo General Academic Hospital with the chief complaint of odynophagia (painful swallowing). The odynophagia had commenced three days prior to his hospital visit and had progressively worsened over the course of one day. The patient experienced intense pain and a burning sensation in the mouth and throat when attempting to swallow, describing the sensation as akin to being cut. He was unable to swallow both solid food and liquids, including saliva, and opening his mouth widely exacerbated the pain. The onset of swallowing difficulty occurred suddenly without any identifiable trigger. Initially, the patient sought care at a private clinic where he received prescribed medications, including antibiotics (i.e., ciprofloxacin), painkillers (i.e., mefenamic acid), mouth drops, and eye drops. Although the symptoms were temporarily alleviated, they subsequently recurred, prompting his admission to Dr. Soetomo General Academic Hospital. Alongside odynophagia, the patient simultaneously exhibited hypersalivation (Figure 1) and excessive tearing (hyperlacrimation). Additionally, non-pruritic reddish rashes suddenly appeared on both hands and feet while in the Emergency Unit. The patient denied any past or present history of genital ulcers, fever, cough, dyspnea, hypertension, or heart disease. He was unaware of any previous history of diabetes mellitus and had not previously received oral antidiabetic medications or insulin. The patient denied any history of oral ulcers, trauma, or contact with rusty objects. He revealed a history of oral narcotics consumption (specifically, trihexyphenidyl) a decade ago. Furthermore, the patient, a married snail retailer of ten years with three children, denied engaging in risky sexual activities, same-sex relationships, or the use of shared injection needles.

**Figure 2:**
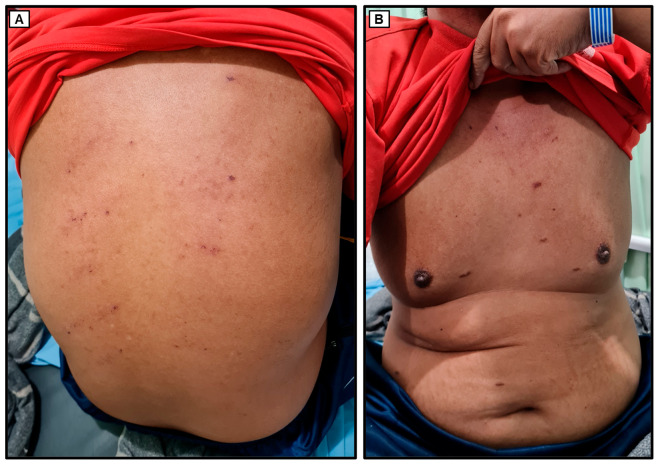
The physical appearance of the patient. No visible rashes or skin lesions were observed on the back of the body (**A**), trunk (**B**), and abdomen (**B**).

Upon the initial physical examination at the Emergency Unit of Dr. Soetomo General Academic Hospital, the patient was found to be seriously ill but alert (*compos mentis*). His vital signs indicated a blood pressure of 129/87 mmHg, a pulse rate of 106 beats per minute, a respiratory rate of 18 breaths per minute, and a body temperature of 36.3 °C. The patient weighed 70 kg and measured 165 cm in height, resulting in a body mass index (BMI) of 25.7 kg/m^2^, classifying him as overweight. Examination of the head revealed bilateral watery red conjunctivae with swollen palpebrae, non-icteric sclerae, and isochoric pupils. The tonsils appeared red and were covered with white plaques. The throat exhibited severe inflammation, with the hard and soft palates covered by thick, malodorous white plaques (Figure 1). The lower lip was swollen and covered with purulent white saliva. No venous dilation was observed in the neck area, and the trachea was centrally located. Jugular venous pressure was within normal limits. Chest examination (Figure 2) showed symmetrical chest expansion, with normal left and right lung fremitus. Auscultation revealed clear breath sounds in both lungs, without rales or wheezing. A cardiac examination did not reveal any visible or palpable *ictus cordis*. The left border of the heart was located at the 4th intercostal space, 1 cm lateral to the left mid-clavicular line, and the right border was at the 4th intercostal space along the right sternal line. The first and second heart sounds were regular, without murmurs or gallops. Abdominal examination revealed a convex abdomen (Figure 2). Bowel sounds were normal upon auscultation. No tenderness, hepatosplenomegaly, palpable masses, or kidney ballottement were detected. Abdominal percussion produced tympanic sounds throughout the abdominal area. Both upper and lower extremities were warm and dry, with no signs of edema. Multiple reddish rashes of varying sizes were observed on both palms and the soles of the feet (Figure 1). No palpable masses were found in the body. Additionally, a painless indurated ulcer was observed adjacent to the penile glans (Figure 1), and a tattoo was present on the right upper arm.

**Figure 3:**
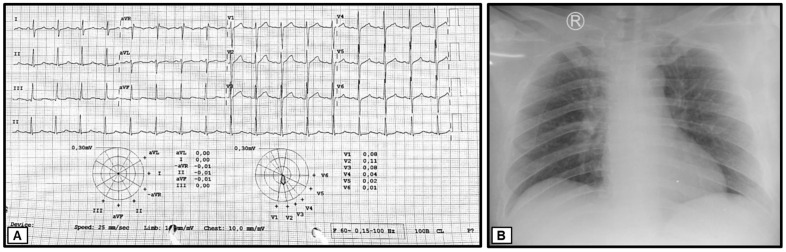
(**A**) Electrocardiographic data showing normal results and (**B**) chest X-ray indicating right hilar lymphadenopathy.

The preliminary laboratory examination conducted in the Emergency Unit revealed the following results: a hemoglobin concentration (Hb) of 18.2 g/dL, hematocrit (HCT) of 52.9%, leukocyte count of 10,000/μL with 64.9% neutrophils and 17.7% lymphocytes, and a platelet count of 298,000/μL. The random blood glucose (RBG) level was measured at 322 mg/dL, blood urea nitrogen (BUN) at 12.7 mg/dL, serum creatinine at 1.0 mg/dL, serum glutamic oxaloacetic transaminase (SGOT) at 16 U/L, serum glutamic pyruvic transaminase (SGPT) at 10 U/L, total bilirubin at 1.3 mg/dL, and direct bilirubin at 0.6 mg/dL. The serum albumin level was 4.18 g/dL, with sodium at 132 mmol/L, potassium at 3.5 mmol/L, and chloride at 94 mmol/L. Furthermore, the rapid human immunodeficiency virus (HIV) test, Hepatitis B surface antigen (HBsAg), and Hepatitis C virus antibody (anti-HCV) results were non-reactive. A chest X-ray examination revealed the thickening of the right hilum, indicating right hilar lymphadenopathy (Figure 3).

The patient was diagnosed with odynophagia due to suspected secondary syphilis, T2DM without signs of hyperglycemic crisis, suspected oral candidiasis, suspected candidiasis esophagitis, mucositis, hypotonic hypovolemic hyponatremia, hyperbilirubinemia, low intake, and hemoconcentration resulting from moderate dehydration. Following the diagnosis, the patient was admitted and a treatment plan was initiated, which included intravenous fluid therapy totaling 1500 mL every 24 h. Additionally, the patient was prescribed a specially formulated nutritional milk-based drink for individuals with diabetes of 200 mL every 4 h, intravenous ceftriaxone (1 g) every 12 h, subcutaneous rapid aspart insulin (4 units) every 8 h before meals, oral ketoconazole (200 mg) daily, oral paracetamol (500 mg) three times a day, nystatin oral drops (4 drops) every 6 h, and antiseptic mouthwash every 8 h. The patient was referred to the Ear, Nose, and Throat (ENT) department for nasogastric tube (NGT) placement, but the procedure failed due to the patient experiencing pain and pulling the NGT, preventing the continuation of the procedure. Subsequently, the patient refused when a second attempt to insert the NGT was made.

Further diagnostic investigations were planned to confirm the diagnosis of secondary syphilis, including Venereal Disease Research Laboratory (VDRL)/rapid plasma reagin (RPR) and Treponema pallidum hemagglutination (TPHA) tests. Screening for autoimmune factors, such as C3, C4, and anti-nuclear antibody (ANA) tests, and infectious diseases that could be falsely identified as or coexist with syphilis, including HIV infection, were also conducted. The patient’s blood glucose profile was evaluated through fasting blood glucose, postprandial blood glucose, and HbA1c tests. Moreover, bacterial and fungal staining and culture were performed to identify the underlying microorganisms contributing to the white plaques observed in the oral cavity.

## 3. Disease and Treatment Progression

### 3.1. In-Hospital Follow-Up Day 2

On the second day of hospitalization, the patient continued to complain of hypersalivation, hyperlacrimation in both eyes, as well as severe odynophagia. The patient also appeared weak and irritable. On examination, the patient’s blood pressure increased to 142/102 mmHg with a heart rate of 113 beats per minute. Laboratory results on the second day revealed a hemoglobin level of 17.3 g/dL, hematocrit of 49.4%, leukocyte count of 6660/μL with 59.3% neutrophils and 18.3% lymphocytes, and a platelet count of 274,000/μL. The erythrocyte sedimentation rate (ESR) was 32 mm, with an HbA1c level of 11.3%, and a RBG level of 350 mg/dL. The patient’s uric acid level was above the normal range (8.6 mg/dL), calcium level was 9.8 mg/dL, magnesium level was 2.06 mg/dL, sodium level was 128 mmol/L (corrected sodium for hyperglycemia 132 mEq/L), potassium level was 3.4 mmol/L, chloride level was 84.0 mmol/L, and serum phosphate level was 3.74 mg/dL. An infection marker examination using procalcitonin showed a result of 0.23 ng/mL, indicating a local infection. The autoimmune panel tests, such as the ANA test, C3, and C4, were within normal limits, while the VDRL/RPR test was reactive (1:32), prompting further testing with TPHA to confirm the diagnosis of secondary syphilis. The patient was prescribed long-acting detemir insulin at a subcutaneous dose of 10 units in the morning (10-0-0 units SC), allopurinol 100 mg once daily at night, and prepared for a single-dose intramuscular injection of benzathine penicillin G 2.4 million units as the treatment for syphilis.

### 3.2. In-Hospital Follow-Up Day 3

On the third day of treatment, the patient’s blood pressure rose to 162/99 mmHg with a heart rate of 112 beats per minute. Additionally, fasting blood glucose was measured at 210 mg/dL, and postprandial blood glucose at 224 mg/dL. Urinalysis results showed +4 protein, +4 glucose, +3 ketones, +3 erythrocytes, and +1 leukocytes, with no bilirubin and nitrite present. Urine albumin levels exceeded the measurable range of the instrument (‘OVER’). The patient was then prescribed oral candesartan (16 mg) once daily in the morning to manage their hypertension while maintaining the rest of the existing medications. The examination conducted by the Dermatology and Venereology department revealed skin and mucosal lesions indicative of Steven-Johnson syndrome (SJS)/toxic epidermal necrolysis (TEN) due to the use of ciprofloxacin and mefenamic acid, with an additional diagnosis of secondary syphilis. The Dermatology department recommended the administration of cetirizine (10 mg) once daily, intravenous injection of dexamethasone (5mg/mL) 1-0-1 (equivalent to prednisone 1mg/kgBW, body weight 60 kg), compress with NaCl 0.9% for 10–15 min three times a day on the lip area, and application of kenalog cream twice daily on the lip area.

### 3.3. In-Hospital Follow-Up Day 4

After the administration of candesartan, the patient’s blood pressure decreased to 146/102 mmHg with a heart rate of 97 beats per minute. Due to extensive white plaques inside the oral cavity, suspected to be oropharyngeal candidiasis (i.e., an opportunistic infection), as well as the presence of risk factors (e.g., tattoo and sexually transmitted infection), the HIV status and cluster differentiation (CD)4 count were evaluated to determine the presence of HIV infection in the patient. HIV testing using one method yielded a non-reactive result, and the CD4 count was within the normal range (696 cells/μL or 35.14%). Meanwhile, TPHA testing showed a positive result of 1/2560, confirming the patient’s syphilis diagnosis. The patient was then given a single-dose intramuscular injection of benzathine penicillin G (2.4 million units). To achieve better blood glucose control, the dose of detemir insulin was increased to 18 units in the morning (18-0-0 units SC), while the dose of aspart insulin was temporarily maintained.

### 3.4. In-Hospital Follow-Up Day 5

On the fifth day of hospitalization, the patient’s complaints did not improve, and he still had difficulty swallowing due to pain in the mouth area. Swab culture tests for bacteria and fungi conducted on the second day of treatment showed no presence of bacteria or fungi in the samples taken from the hard palate. However, because there was a decrease in the white plaques in the mouth after the administration of oral ketoconazole, since the first day of treatment and clinically, candidiasis was still possible in this patient due to his T2DM. The oral ketoconazole was switched to intravenous fluconazole (200 mg) once daily. Additionally, despite the increase in insulin dose on the previous day, there was no significant change in the random blood glucose (RBG 256 mg/dL). Therefore, the patient was prescribed oral metformin (500 mg) every 8 h to improve possible insulin resistance in this patient, and the dose of detemir insulin was increased to 26 units in the morning (26-0-0 units SC). For the erosion on the lips, the patient was advised by the Dermatology department to topically apply 0.9% NaCl for 10–15 min, 2–3 times a day.

### 3.5. In-Hospital Follow-Up Day 6

The patient complained of increasing oral pain, and during the local examination of the mouth, reddened mucosa with reduced white plaques was observed. To rule out the suspicion of oral lesions caused by drug eruptions, such as in SJS, a serum CD8 examination was conducted, revealing results within normal limits (CD8 467 cells/μL or 22.83%). This finding, together with dissimilarities to the clinical presentations of SJS [10], suggest that CD8+ T cell-mediated type IVc hypersensitivity reactions, such as in SJS or TEN, were not likely the cause of these lesions. Moreover, from the medical history, drug consumption occurred after the onset of the initial lesions, making it less likely to be the trigger for the oral mucosal lesions. HIV infection has also been ruled out through non-reactive results from testing using three different methods and the negative HIV viral load. Consultation with the Ophthalmology department revealed acute blepharoconjunctivitis in both eyes, and the patient was prescribed additional medication in the form of levofloxacin eye drops, one drop every 4 h for both the right and left eyes. Due to uncontrolled blood glucose levels, the dose of aspart insulin was increased to 8 units every 8 h before meals.

### 3.6. In-Hospital Follow-Up Day 7 until Discharge

**Figure 4:**
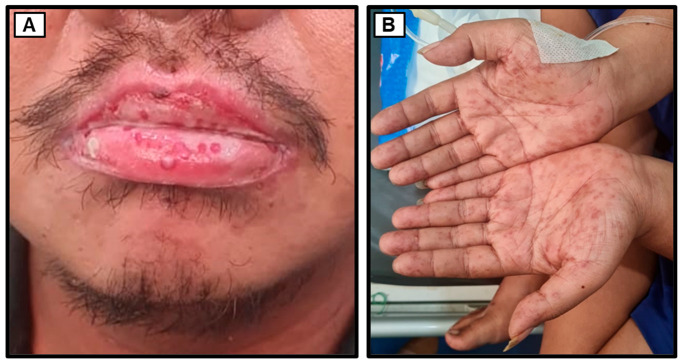
The physical appearance before discharge. (**A**) The edema on the lower lip has significantly reduced, and the blepharoconjunctivitis has been resolved. (**B**) The palmar rashes were still present at the point of discharge.

**Figure 5:**
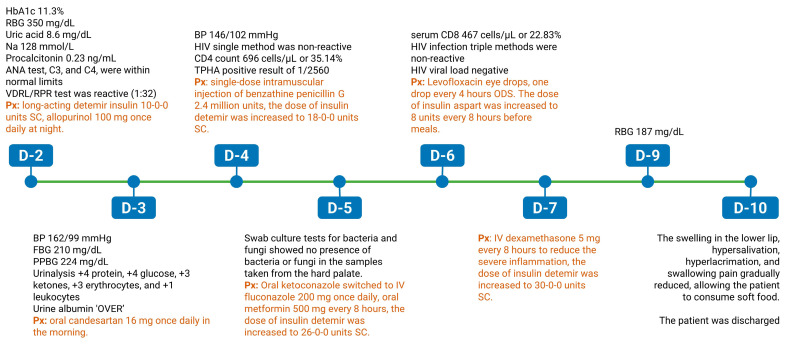
The timeline of disease and treatment progression. This timeline displays the key clinical, laboratory, and microbiological findings during the in-hospital treatment of the patient.

On the 7th day of treatment, the patient continued to complain of hypersalivation and severe pain upon swallowing, although the patient was able to drink milk. Therefore, we administered intravenous injections of dexamethasone (5 mg) every 8 h to reduce the severe inflammation. To anticipate potential spikes in the patient’s blood sugar levels, which could lead to a hyperglycemic crisis, the dose of detemir insulin was increased to 30 units subcutaneously in the morning (30-0-0 units SC). As a result, on the 9th day of treatment, the patient’s RBG decreased to 187 mg/dL. We also performed a re-evaluation of the BUN/SC levels, and they were within the normal range (BUN 20.8 mg/dL and SC 0.8 mg/dL) with an eGFR of 125 mL/min. By the 10th day of treatment, the swelling in the lower lip, hypersalivation, hyperlacrimation, and swallowing pain gradually reduced (Figure 4), allowing the patient to consume soft food bit by bit. Since the patient’s overall condition had improved, on the 10th day of treatment, he was allowed to be discharged from the hospital and continue further treatment as an outpatient. We also planned for the patient’s spouse to undergo examinations regarding the potential transmission of syphilis to/from the patient. Figure 5 displays the timeline of disease progression and treatment of the patient. 

## 4. Discussion

Examinations using darkfield microscopy and molecular tests to detect *Treponema pallidum* in exudates or tissues are considered the gold standard methods for diagnosing syphilis, particularly in the presence of active *ulcus durum* or condyloma lata [11,12]. However, these techniques are not always clinically feasible and may not be readily available, particularly in healthcare facilities in developing countries where syphilis prevalence is high. The United States Center for Disease Control and Prevention (CDC) recommends that a presumptive diagnosis of syphilis can be established by employing two serological laboratory tests: nontreponemal VDRL/RPR and treponemal tests (such as rapid treponemal assays, TPHA, enzyme immunoassay, chemiluminescence immunoassays, and immunoblot) [12]. 

**Figure 6:**
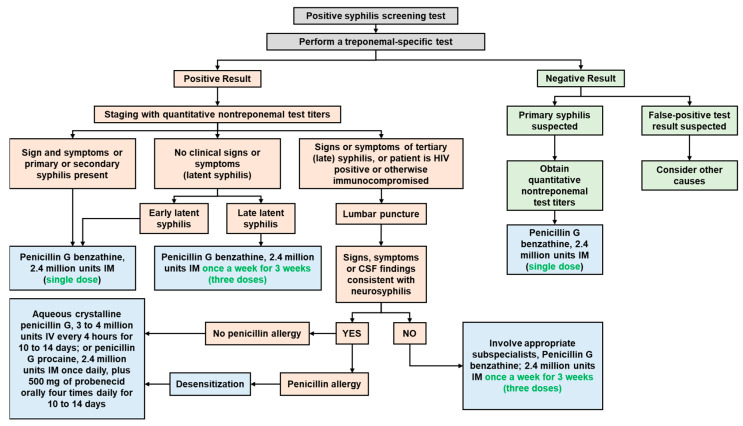
The algorithm for syphilis diagnosis and treatment according to the American Academy of Family Physicians (AAFP) (CSF = cerebrospinal fluid; HIV = human immunodeficiency virus; IM = intramuscular; IV = intravenous) [11].

When the body is infected with syphilis, it generates non-specific antibodies that bind with cardiolipin [11,13]. This interaction is the basis for conventional nontreponemal tests such as VDRL and RPR. However, due to their lack of specificity, these tests can yield false-positive results due to factors like infections, autoimmune disorders, vaccinations, drug use, pregnancy, and age. Conversely, in situations where a high concentration of antibodies is present (as in undiluted samples), they might hinder the formation of antigen-antibody complexes, potentially leading to false-negative results [11,12]. Antibody titers from the nontreponemal test could potentially indicate disease activity and are employed to track the effectiveness of treatment. Nontreponemal test titers may decrease after treatment, but inadequate serologic response or persistent antibodies (i.e., serofast reaction) occur in some cases. Presumptive treatment is recommended when serologic tests do not align with clinical findings, especially for individuals at risk. To confirm syphilis diagnosis, individuals with a positive nontreponemal test (Figure 6) should undergo a treponemal-specific test [11,12]. Treponemal-specific tests identify antibodies targeting specific components of *Treponema pallidum* [11]. These tests are mainly employed to validate the diagnosis of syphilis in individuals who have tested positive on a nontreponemal test. Reactive treponemal tests often remain positive for life, except for 15–25% of patients treated in the primary stage who become nonreactive after 2–3 years. Treponemal antibody titers cannot predict treatment response and should not be used for this purpose [12].

In this case, during the physical examination, we observed multiple rashes on the palms and soles, lesions on the oral mucosa and conjunctiva, as well as painless (indurated) ulcers on the penile glans. Therefore, we suspected that the patient might be suffering from secondary syphilis. Subsequently, the nontreponemal method VDRL/RPR and treponemal-specific TPHA were employed to establish the clinical diagnosis of secondary syphilis. The results showed an increase in VDLR/RPR titer to 1:32 and TPHA titer of 1/2560, confirming the diagnosis of syphilis. Until the end of the treatment period, the patient was unwilling to acknowledge risky sexual behavior, which could explain the source of transmission. Therefore, an examination of the patient’s spouse is scheduled at the outpatient clinic during the patient’s follow-up appointment.

**Figure 7:**
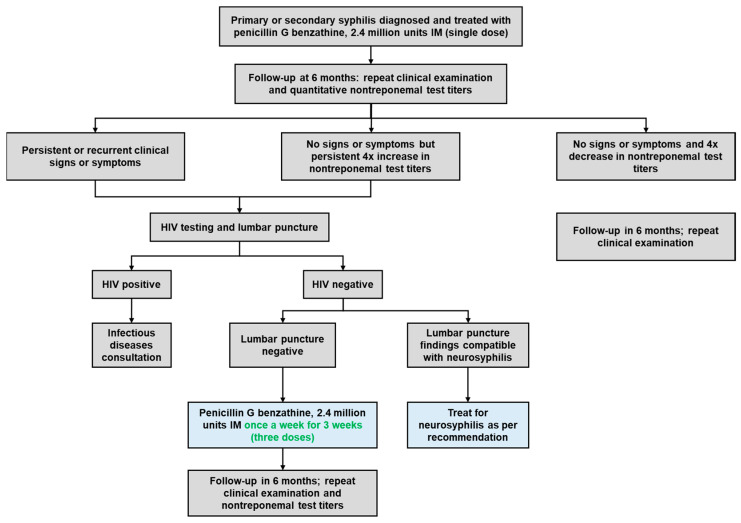
The follow-up algorithm for patients with primary or secondary syphilis is based on the American Academy of Family Physicians (AAFP) guidelines. [11].

Once the clinical diagnosis of syphilis is confirmed, in line with CDC guidelines, parenteral penicillin G is the primary and preferred treatment. The choice of preparation (such as benzathine, procaine, or aqueous crystalline), dosage, and duration of treatment are contingent upon the disease stage and clinical symptoms (see Figure 7). Late latent syphilis (lasting over one year) and tertiary syphilis demand extended therapy due to the possibility of slower organism division. Individuals with latent syphilis of unknown duration require prolonged treatment to ensure adequate therapy, especially if they may not have contracted syphilis within the past year. An acute febrile reaction known as the Jarisch–Herxheimer reaction may occur in response to antibiotics combating high bacterial loads in the body, especially in early syphilis. However, the occurrence of this reaction should not hinder or delay the initiation of therapy [12]. Following the administration of appropriate treatment, patients should undergo quantitative nontreponemal test titers to assess their response to treatment. Nontreponemal test titers should decrease by at least four times within six months after treating primary or secondary syphilis (Figure 6), and within 12 to 24 months after treating latent or late infection [11].

In this case, after the diagnosis of secondary syphilis was confirmed, a single-dose intramuscular injection of 2.4 million units of benzathine penicillin G was administered, and the patient was observed for the possibility of Jarisch–Herxheimer reaction. Following the injection of benzathine penicillin, the patient experienced myalgia and a mild fever, but these symptoms spontaneously subsided two days after the injection. Oral lesions and hypersalivation started to diminish approximately 4 days after the benzathine penicillin injection. 

The association between syphilis and DM has been a point of debate for more than a century. Necropsy results from six patients with DM revealed signs of syphilis, leading to the conclusion at that time that syphilis was one of the causes of DM [14,15]. A more recent study also reported a higher prevalence of DM in patients with neurosyphilis compared to non-neurosyphilis patients, non-syphilis patients, and healthy individuals [16]. Although the precise mechanisms remain unknown, a bioinformatics study aimed to explore the differentially expressed genes (DEGs) in a rabbit model of syphilis combined with diabetes. The study found that the downregulated DEGs were notably associated with biosynthesis of antibiotics, carbon metabolism, and protein digestion, whereas the upregulated DEGs were primarily linked to cancer and the phosphoinositide 3-kinase (PI3K)-Akt signaling pathway [17]. In the case we presented, the patient was unaware of any previous history of T2DM, making it difficult to establish whether syphilis was the cause or strongly associated with the onset of T2DM in this patient. However, T2DM in this case required a combination of oral antidiabetic medication (i.e., metformin) and insulin at relatively high daily doses. This could be reinforced by the use of intravenous injections of corticosteroids [18,19] and the state of infection [20,21], which further increased the blood glucose levels. Until the end of the treatment period, the patient had not yet achieved the target blood glucose level, despite a significant decrease in blood glucose levels compared to the ones upon hospital admission. 

A previous study showed that in patients with secondary syphilis, there was evidence of low-level spirochetemia and signs of monocyte activation, with no detection of systemic cytokine production. Secondary syphilis patients displayed reduced circulating dendritic cells and interferon (IFN)γ-producing cytotoxic natural killer (NK) cells, along with an emerging CD56^−^/CD16^+^ NK-cell subset in the bloodstream. Skin lesions exhibited macrophages, an increased presence of CD8^+^ T cells compared to CD4^+^ T cells, and enriched CD56^+^ NK-cells. These lesions contained transcripts for various cytokines, chemokines, markers for macrophage and dendritic cell activation, Fc-mediated phagocytosis receptors, and effector molecules associated with CD8 and NK-cell cytotoxic responses [22]. In this case, the serum levels of CD4 and CD8 were within normal limits. This supports the findings of the aforementioned research, indicating that in secondary syphilis, there is no systemic cytokine production, although inflammatory cells can be found in the associated mucocutaneous lesions.

Syphilis, often referred to as the great imitator, can mimic the clinical symptoms of a wide range of diseases, spanning from infectious to autoimmune diseases. For example, oral lesions and odynophagia in syphilis have been reported previously. A case study described a patient who experienced months of odynophagia and recurrent oral lesions. Subsequent endoscopy revealed an esophageal ulcer as the underlying cause of odynophagia. Biopsy, coupled with a fluorescent antibody stain for *Treponema pallidum*, confirmed that syphilis was the culprit behind this lesion. The condition was successfully resolved with an intramuscular injection of penicillin [23]. In this case, severe oral lesions were observed, together with white plaques suggestive of candidiasis. Examination by the Department of Dentistry and Oral Medicine revealed multiple oral ulcers resembling the lesions found in drug eruptions or erythema multiforme. We suspected that the severe inflammation and oral ulcers were the cause of the patient’s sialorrhea and swelling of the lower lip. Following the administration of benzathine penicillin and steroids, the lesions in the oral cavity began to diminish, along with the hypersalivation. Subsequently, the patient was able to consume soft food without experiencing excruciating pain.

## 5. Summary

In this case report, we present a clinical case involving a patient with newly discovered T2DM suffering from secondary syphilis and severe odynophagia. The patient initially complained of intense pain upon swallowing, accompanied by hypersalivation (sialorrhea), increased tear production (hyperlacrimation), swelling in the lower lip, and rashes on the hands and feet. Upon further examination, positive results were obtained for VDRL/RPR and TPHA, with no signs of accompanying blood-borne or sexually transmitted viral infections. The patient was then administered a single-dose intramuscular injection of 2.4 million units of benzathine penicillin G and other supportive medications to improve his condition. Steroid injections (i.e., dexamethasone) were administered at the end of the treatment period to alleviate the severe inflammation occurring in the oral mucosa. By the end of the hospital stay, the patient’s complaints significantly reduced, and he was able to consume soft foods. The patient was scheduled to continue his treatment in the outpatient clinic to identify the source of his syphilis transmission.

## Data Availability

No new data were created.
